# Assessing current capabilities for incorporating lipidomics in multiomics data integration

**DOI:** 10.1093/bib/bbag276

**Published:** 2026-06-04

**Authors:** Dylan H Ross, Raghav Jain, Hyeyoon Kim, Javier E Flores, Soumaydeep Sarkar, Chaevien S Clendinen, Jennifer E Kyle, Tao Liu, Sara J C Gosline

**Affiliations:** Biological Sciences Division, Pacific Northwest National Laboratory, 902 Batelle Boulevard, Richland, WA 99354, United States; Biological Sciences Division, Pacific Northwest National Laboratory, 902 Batelle Boulevard, Richland, WA 99354, United States; Biological Sciences Division, Pacific Northwest National Laboratory, 902 Batelle Boulevard, Richland, WA 99354, United States; Biological Sciences Division, Pacific Northwest National Laboratory, 902 Batelle Boulevard, Richland, WA 99354, United States; Biological Sciences Division, Pacific Northwest National Laboratory, 902 Batelle Boulevard, Richland, WA 99354, United States; Environmental and Molecular Sciences Laboratory, Pacific Northwest National Laboratory, 902 Batelle Boulevard, Richland, WA 99354, United States; Biological Sciences Division, Pacific Northwest National Laboratory, 902 Batelle Boulevard, Richland, WA 99354, United States; Biological Sciences Division, Pacific Northwest National Laboratory, 902 Batelle Boulevard, Richland, WA 99354, United States; Biological Sciences Division, Pacific Northwest National Laboratory, 902 Batelle Boulevard, Richland, WA 99354, United States

**Keywords:** multi-omics integration, lipidomics, pathway analysis, artificial intelligence

## Abstract

Comprehensive analyses of multiple biological components including nucleic acids, proteins, metabolites, and lipids (i.e. “multiomics”) provide unique insights into complex biological processes. Combining insights from these components through multiomics data integration enhances the depth and nuance of biological understanding available from these measurements. Among methods that integrate data across different technologies (e.g. mass spectrometry, sequencing), those that link components based on biological prior knowledge—pathway analyses—represent the most direct way of translating molecular-level observations into meaningful biological insights. However, significant barriers exist that prevent full utilization of metabolomics and especially lipidomics data in pathway integration. Challenges include the fast turnover and complex interactions of small molecules compared to biological macromolecules, low metabolite annotation rates, isomerism among lipids, and a lack of lipid representation in existing pathway knowledge bases. While these issues stem from a variety of causes, improvements to multiomics pathway integration including better incorporation of lipids into pathway knowledge bases and increased adoption of artificial intelligence approaches can greatly enhance the utility of small molecule -omics data, particularly for lipidomics. Here, we describe the current landscape of multiomics integration tools with an emphasis on support for metabolomics and lipidomics data, we highlight their capabilities and gaps with tangible examples using real multiomics data, and provide our perspective on how these approaches can be improved to better support generation of useful biological insights from complex multiomics data.

## Introduction

Diverse biotechnological advances have enabled the measurement of detailed cellular activity and discovery of numerous biological and biomedical findings. DNA sequencing platforms [1, 2] and analysis tools have rapidly accelerated the measurement of complete genomes, leading to the identification of specific cancer-driving mutations [3, 4] and genetic indicators of disease [5, 6]. Likewise, the measurement of complete transcriptomes has enabled the use of RNA as a proxy to protein-level expression, providing deeper insights across human disease (e.g. cancer [7, 8], Alzheimer’s disease [9, 10]) to identify systematic alterations in protein signaling across diseases and tissues. Comprehensive mass spectrometric (MS) analyses have provided orthologous measurements of biomolecules including proteins, posttranslational modifications such as phosphorylation sites, metabolites, and lipids, giving rise to additional atlases of cell-wide activity: the proteome [11], metabolome [12], and lipidome [13], respectively, which have provided numerous biological advances in cancer and metabolic disorders.

**Figure 1:**
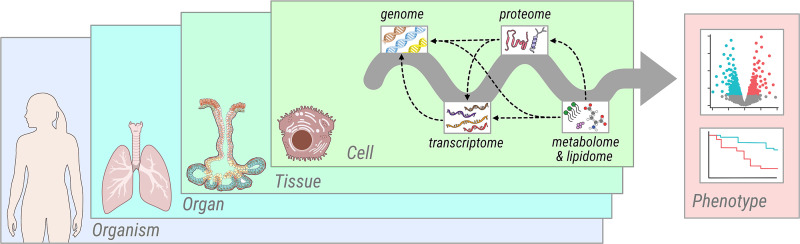
The multiomics data landscape. The solid line indicates the canonical transfer of biological information from genetic/non-genetic sources to phenotype. Dotted lines indicate the known interactions and feedback mechanisms between different biomolecules within a system running counter to the canonical direction.

**Figure 2:**
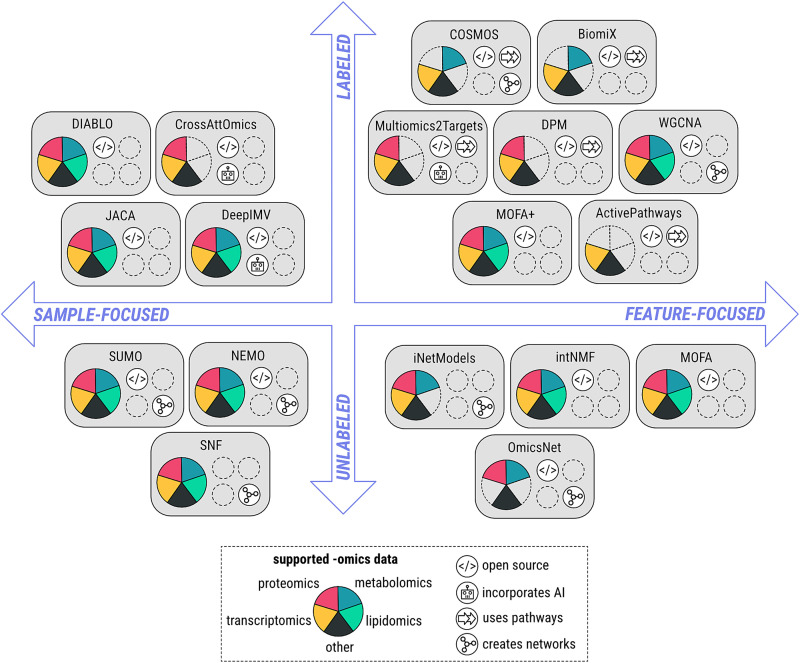
Categorization of selected multiomics data integration tools based on analysis objectives. The position of the tools indicates the primary analysis objectives that they support, according to a 2D categorization scheme that distinguishes sample- versus feature-focused analyses in addition to those that deal with labeled versus unlabeled data. The depiction of each tool reflects the -omics data types that they support, and additional highlighted characteristics of interest including whether the software is open source or incorporates AI in some capacity.

While each -ome on its own can produce useful findings, these individual results represent an incomplete picture of the biological system. Combining measurements of different components from a sample (i.e. multiomics) promises to provide a more complete and nuanced picture of biological processes and their interplay within a larger system (Fig. 1) [14]. Successful application of multiomics relies on advancements in computational tools [15–17] that integrate this multimodal data to identify new and/or complementary features of interest or patterns of expression among related samples, enabling cell-wide modeling of specific perturbations (Fig. 2). A subset of tools of particular interest is those that focus on pathway-based integration: mapping molecules to annotated biological pathways based on prior knowledge to devise mechanistic hypotheses from -omics level data [18]. To date, however, these pathway-based tools have limited or no capability to fully capture and integrate small molecule data from lipidomics and metabolomics measurements [19–22]. This is largely due to gaps in existing data resources: pathway enrichment tools rely on knowledge of which molecules physically interact with each other to carry out a biological function; this data, with few exceptions (cite KEGG), is absent for small molecules.

Another major challenge of pathway analysis of small molecules lies in their inherent nature: lipids and metabolites are chemically diverse and lack a genetically encoded blueprint for identification, such as a known amino acid or nucleic acid sequence. This structural complexity results in largely incomplete database to support feature annotation [23] leading to a significant amount of metabolite “dark matter” (molecules not yet mapped) [24]. There is also the added difficulty that some metabolites fall on multiple pathways or have not yet been mapped to pathways (as is especially the case for lipids [25]). Despite these challenges, small biomolecule -omics provides a direct window into the dynamic processes of metabolism, including signaling and cellular regulation, that cannot be fully captured by proteogenomic data alone [26]. This noticeable gap in small molecule pathway integration comes at a time when the role of these molecules in disease has been highlighted in numerous studies [27–31].

In this review, we highlight the challenges of incorporating metabolomics and lipidomics data for multi-omic pathway integration. We (i) review of the current landscape of strategies and tools for integrating small biomolecule -omics data; (ii) describe the gaps and limitations among existing methods, chief among which is in the incorporation of small molecule data with other -omes using biological context using pathways; (iii) introduce a novel classification scheme for integration tools oriented around practical analysis objectives while grounding our discussion using tangible multi-omics data analyses examples, and lastly; (iv) share our perspective on future solutions to improve algorithms supporting biological interpretation, including the promise of artificial intelligence (AI) in this domain.

## The landscape of multiomics data integration tools is diverse and supports a variety of analysis objectives

The general purpose of multiomics data integration is to combine observations of different biological components from a sample in order to build a more complete picture of its overall biological state. To achieve the end goal of improved biological understanding, we classify multiomics integration tools as supporting either sample-focused or feature-focused analysis objectives (Fig. 2). This categorization diverges from previous reviews [17, 32–35] that have classified multiomics integration methods based on the mechanics of the approach or algorithmic details (e.g. concatenation-based, transformation-based, and model-based). In this scheme, sample-focused methods demonstrate capabilities primarily aligned with sample-level analyses, such as fitting models predictive of a particular sample property (e.g. disease status) or identifying latent groupings of samples that might be tied to domain-relevant classifications (e.g. cancer subtypes). Broadly, these methods answer the question: “how are my samples different from one another?”. Conversely, feature-focused methods are better suited for characterizing inter-feature relationships, such as through network or pathway analyses, answering the question: “what processes are changing in my samples beyond expected noise?”. Both sample- and feature-focused methods can be matrixed further into labeled and unlabeled approaches, subcategories distinguished by whether samples are assigned labels reflecting a trait of interest (e.g. treatment or control) and those labels are taken into consideration during data analysis [36, 37]. While these categorizations are not mutually exclusive of one another, they serve as a useful guide for understanding the particular types of problems a tool is best suited to solve.

### Sample-focused integration predicts biological states

The main objective of sample-focused integration methods is to determine multiomic features that can explain and predict differences between biological states that are associated with an external trait of interest (labeled data) or for grouping samples based on shared characteristics (unlabeled data). In either case, construction of these signatures tends to prioritize sparsity and predictive power over interpretability. These methods are applied when the objective is to robustly predict an external trait of interest or to assign a sample to a group or subtype using a small number of important features.

DIABLO [38, 39], JACA [40], DeepIMV [41], and CrossAttOmics [42] (Fig. 2, upper left) are computational methods designed to integrate labeled multi-omics datasets to uncover meaningful biological insights while supporting biomarker discovery and predictive modeling. All four approaches share the overarching goal of identifying cross-omics relationships, enabling the integration of diverse biological layers to reveal correlated features associated with a trait of interest. Though each employs distinct algorithmic frameworks—ranging from generalized dimension reduction to deep learning architectures—their differences primarily lie in implementation focus: DIABLO emphasizes flexibility and interpretability for exploratory analyses, JACA provides a statistically unified framework for robust classification and association modeling, DeepIMV leverages nonlinear modeling for accommodating missing data and handling large-scale omics studies, and CrossAttOmics uses a cross-attention mechanism to capture interactions between components with known regulatory links. These methods offer a practical means of predicting traits of interest from multiomics data, and the compact multi-component signatures that form the basis of these predictions can additionally provide some insights into the systems that underly the predicted biological states.

Unlabeled multi-omics data integration methods focus on characterizing sample-level associations using trends in the high-dimensional data without relying on external labels or predefined traits. Similarity Network Fusion (SNF) [43], SUMO [44], and NEMO [45] exemplify this approach (Fig. 2, lower left), each aiming to uncover shared patterns and clustering structures across omics datasets. While all three methods integrate sample–sample similarity matrices to reveal cross-omics relationships, they differ in how they combine information. SNF iteratively reinforces consistent signals using a nonlinear combination method [46] to construct a single similarity network that can be clustered. SUMO leverages symmetric matrix factorization to learn shared low-dimensional representations for robust cross-omics clustering. Meanwhile, NEMO emphasizes local neighborhood relationships, using relative similarity scores and simple averaging for integration. Together, these methods offer the ability to perform exploratory analysis of multiomics data at the sample level without relying on labeled data, revealing groups of related samples and the features that drive those groupings.

### Feature-focused integration contextualizes changes across components

Feature-focused multi-omics integration methods aim to place the molecular measurements in a biological context across samples by identifying shared patterns and sources of variation among features. This process may be guided by external traits or known sample groups (labeled data), or it can be used to find new relationships between samples (unlabeled data). These feature-focused analyses tend to prioritize contextualizing important features over sparsity or outright predictive power, and are therefore best suited to analysis objectives including mechanistic interpretation, hypothesis generation, or expansion of biological knowledge.

Feature-focused analyses are typically applied to labeled data, where the objective is to identify the biological processes that are altered with respect to some trait of interest. Integration tools in this category (Fig. 2, upper right) frequently leverage inter-feature associations (known relationships between molecules in the form of a network) and/or prior knowledge (list of molecules belonging to pathways) in order to broaden the biological context around important features, supporting deeper biological interpretation and hypothesis generation. Weighted Gene Correlation Network Analysis (WGCNA) [47] constructs feature-level networks based on pairwise correlations where the pairwise relationship between features is determined by correlation. As a result, WGCNA identifies highly similar groups of features, summarizing them with “eigengenes” to model their associations across datasets. Multiomics2Targets [48] packages together existing tools for performing enrichment analyses on phosphoproteomics (measurement of active phosphorylation site on proteins), proteomics, and transcriptomics data from the same samples, and uses a large language model (LLM) to summarize and interpret analysis results into an output report, with a focus on application to studying human cancers. ActivePathways [49] combines statistical significance from multiple omics datasets to identify and prioritize enriched pathways, represented by lists of features, using a ranked hypergeometric test, highlighting pathways found uniquely through data integration for visualization and further exploration. Directional P-value Merging (DPM) [50] integrates statistical evidence across omics datasets by combining *P*-values while accounting for the expected direction of molecular changes, highlighting functionally coherent entities like genes or pathways. COSMOS [51] incorporates biological knowledge into network-level reasoning to causally connect deregulated elements, including transcription factors, metabolites, and kinases, within prior knowledge networks (e.g. known associations between molecules such as physical interactions), enabling contextualization of sample-specific changes. MOFA [52] and MOFA+ [53] generalize principal components analysis into a multi-omics framework, uncovering latent factors that capture shared and unique sources of variation across datasets, supporting applications such as trait associations (labeled data, MOFA+) and clustering (unlabeled data, MOFA). BiomiX [54] is a tool that integrates single-omics and multi-omics data using MOFA to capture sources of variation, optimize factor selection, perform pathway analysis, annotate factors with PubMed-assisted literature searches, and provide user-friendly interactive data visualization and flexible pipeline settings. This category is particularly rich with tools because understanding feature-level associations is a central analysis objective across many multiomics studies. The diversity of tools within this category reflects the broad range of solutions that have been developed to tackle a common problem, with each tool prioritizing different aspects of integration and interpretation to meet specific scientific needs and goals.

Feature-focused methods can also be applied to unlabeled data, where the general analysis objective is to derive cross-omics profiles of features that are characteristic of different groups of samples without knowing anything about the samples *a priori*. OmicsNet 2.0 [55] integrates multiomics data to create and refine biologically relevant networks, offering advanced visual analytics in 2D/3D, support for diverse omics types like SNPs, and enhanced reproducibility through incorporation of other R-based tools and collaborative sharing features. iNetModels [56] is a web-based platform that integrates tissue- and cancer-specific gene co-expression networks (GCNs) with personalized multi-omics biological networks (MOBNs), providing tools for interactive visualization, identifying functional relationships, uncovering biomarkers, and validating clinical findings. intNMF [57] is an integrative clustering method based on nonnegative matrix factorization (NMF) that classifies samples into distinct subtypes by integrating multiple molecular data types without relying on distributional assumptions, offering robust and flexible subtype discovery with user-defined weights. These methods are particularly useful for exploration of complex multiomics datasets, without the need for predefined sample labels or associated traits.

### Example multiomic analysis highlights sample- and feature-focused insights and limitations

To compare the abilities of these tools to analyze and contextualize multiomics measurements that contain lipids and metabolites together with other molecules, we analyzed the same data using both a sample-focused tool, DIABLO, and a feature-focused tool, COSMOS. The data we used was comprised of transcriptomic, proteomic, phosphoproteomic, lipidomic, and metabolomic data from the Clinical Proteomic Tumor Analysis Consortium (CPTAC) acute myeloid leukemia (AML) study [58]. This study analyzed peripheral blood and/or bone marrow samples from 84 patients representing 2 classifications of AML maturation states [59], committed (*n* = 41) or primitive (*n* = 43), which have been shown to drive prognosis in most patients and exhibit a strong set of transcriptional markers that can be used to assign maturation state to unseen patient samples. In this study, we expanded upon our initial work by showing how multiomic integration tools can either (i) better classify samples by maturation state (using DIABLO as an exemplar of sample-based approaches) or (ii) identify features and pathways that determine maturation state (using COSMOS as our feature-based approach).

**Figure 3:**
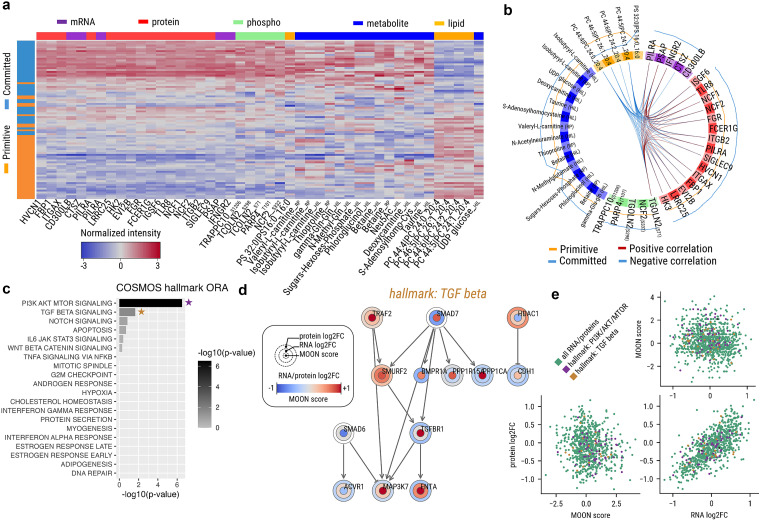
Results from DIABLO and COSMOS analysis of example multiomics dataset. (a) Heatmap showing normalized abundances of features selected for training the DIABLO model. The features (columns) are colored according to the -omics dataset they come from and the samples (rows) are colored according to their corresponding tumor classification (primitive or committed). (b) Circos plot showing correlation between features selected for training DIABLO model. Each group of features is colored according to the -omics dataset they come from. The lines along the outer perimeter indicate average relative abundance in primitive and committed tumor classification groups, and interior lines indicate features with a high degree of positive or negative correlation. (c) Results from hallmark ORA performed using genes included in the MOON analysis result network. The top two identified hallmark pathways with *P*-values <.05 are highlighted with stars. (d) Visualization of subsets of the MOON analysis result network from the highlighted pathway: TGF beta signaling. Round nodes represent proteins, which may have corresponding transcriptomics/proteomics log2FC values and an associated MOON score, which is mapped to the centermost circle. Log2FCs from transcriptomics and proteomics datasets, where available, are mapped to outer concentric rings. All log2FC and MOON scores are mapped to the same color scale as indicated by inset legend. Graph edges depict activation and inhibition relationships between nodes as specified in the legend. (e) Scatter plots comparing transcriptomic/proteomic log2FC versus MOON score from complete filtered PKN. Colors indicate subset of features, as described by legend inset.

In DIABLO analysis, the expected strength of associations between different omics types is a user configurable model parameter, via a so-called “design matrix.” For our analysis, we prepared a design matrix to connect the individual -omics data (transcripts, proteins, phosphosites, metabolites, and lipids) according to their observed covariances, as determined by an initial partial least squares (PLS) regression analysis. We performed automated parameter tuning with cross validation to determine the optimal number of components to use in the final DIABLO analysis and how many features to retain from each -omics type. Ultimately, a final model was trained using a single component consisting of 5–15 features from each -omics data type, which was capable of predicting committed versus primitive tumor subtype with an area under the receiver operating characteristic curve (ROC AUC) of >0.95. The impressive classification performance of the model using only a tiny subset (<0.09%) of the original multiomics features is a testament to the power of sample-focused analyses to distill complex datasets down to a highly informative subset that is useful for robustly predicting biological states. A secondary benefit of this analysis is the feature-level insights, which can provide some clues as to the broader biological context underlying the predicted biological states. Figure 3A depicts the selected multiomic features (columns) and their normalized abundances from the samples (rows) colored according to primitive or committed tumor classification. From these features, we see a highly correlated abundance profile across the mRNA, protein, metabolite, and phosphosite features selected, where these features are up-regulated in the committed samples, while the lipids selected are anti-correlated and down-regulated in the same patients (Fig. 3A). This pattern is further exemplified by the correlation pattern across features depicted in the circos plot in Fig. 3B, where mRNA, proteins, and phosphosites are highly correlated with each other and lipids are anti-correlated. Interestingly, we fail to identify any significant correlation between the metabolomic features and other types of features, reinforcing their scattered abundance profiles observed in the heatmap. The sparsity of this composite signature makes it difficult to discern any higher-level insights into the biological systems that are altered between tumor classifications, requiring a feature-by-feature search of the results. In doing so, one can find evidence of disease relevance in diacylglycerophosphocholines (PCs) containing arachidonic acid (FA 20:4) suggesting an alteration in the outer membrane fluidity and/or an increase in fatty acid precursors with immune-related functions, and ITGB2, which has been found as a transcript that correlates with patient outcome in AML [60].

To compare these results to a more feature-focused tool, we analyzed transcriptomic, proteomic, and metabolomic data from the same AML cohort using COSMOS (Fig. 3C–E). COSMOS does not support lipidomics data so they were not included in this analysis. We employed the metafootprint (MOON) function in COSMOS that infers transcription factor activities based on transcript abundances, maps these inferred activities onto a prior knowledge network (PKN) along with transcriptomic and metabolomic inputs, and then filters and scores based on coherence between information in the PKN, inferred activities, and observed abundances. Ultimately, the MOON analysis produces a filtered subset of the PKN, which is represented by node attributes and signed interactions. Among these node attributes is a MOON score, which reflects consistency between the sign of interactions in the PKN, inferred activities, and observed abundances both up- and downstream of a given node with the sign reflecting regulation in the up (+) or down (−) direction. We used this filtered subset of the PKN to perform overrepresentation analysis (ORA) for hallmark pathway genes [61] against a human proteome background with BioMart [62], which highlighted two significantly enriched pathways: PI3K AKT MTOR signaling and TGF beta signaling (Fig. 3C). The PI3K/MTOR pathway activity confirms the findings from the original study and is well known to be aberrantly activated in AML [63]. TGF beta signaling also has promising biological relevance as it has been shown to be associated with greater degrees of AML progression [64]. However, when we examine the feature-specific results (Fig. 3D), we see that the MOON scores do not always agree well with the observed protein/transcript abundances—sometimes disagreeing entirely—drawing into question whether the MOON analysis may be obfuscating, rather than summarizing, what is happening with pathways at the individual protein level. Indeed, bulk comparison of MOON scores against RNA or protein Log2 fold-changes showed no discernable correlations despite the apparent correlation between RNA and protein fold-changes (Fig. 3E). While most models assume that there is some noise in the high-throughput measurements, we expect systematic results to generally align with the -omics readouts. We additionally note that there are no metabolites present in the highlighted pathway; therefore, the metabolomics data were underutilized in this example analysis. This demonstration produced high-level insights into biological processes that may be altered between primitive and committed AML, but understanding how the system changes between these states at a more granular level would require further analyses.

By comparing examples of the sample- and feature-focused categories of multiomics integration tools, we can see the distinct types of insights they provide. DIABLO identifies the minimum features needed to confidently classify samples as “Primitive” or “Committed” using each multiomics data type. While these features offer hints about higher-order processes altered between sample groups, their sparsity limits the ability to interpret biological significance solely from this analysis. In contrast, the feature-focused COSMOS analysis prioritized biological interpretation by contextualizing interactions between features within a framework of prior knowledge, facilitating hypothesis generation. This reliance on prior knowledge, however, also creates the potential for overlooking important features that are not yet represented in the knowledge base. We also found that the MOON analysis has the potential to obfuscate the overall up- or down-regulation of a pathway of interest. Together, these examples highlight the complementary strengths and inherent limitations of sample- and feature-focused multiomics integration tools, underscoring the importance of aligning tool selection with data analysis objectives.

### Pathway analysis and networks apply statistical rigor to biological hypotheses

An important capability among -omics integration tools is aligning features to known biological networks or pathways, collectively termed functional analyses. These functional analyses represent a critical link between statistical observations made at the level of the omics dataset and meaningful biological hypotheses based on mechanistic understanding of the biological system [18, 22, 65]. As such, there are a wealth of databases available for mapping unknown features such as KEGG [66], The Gene Ontology (GO) [67], the molecular signatures database (MSIGDB) [61], LIPID MAPS [68], SwissLipids [69], and the human metabolome database (HMDB) [70], as well as tools that can leverage these databases to identify pathways such as Gene Set Enrichment Analysis (GSEA) [71], enrichR [72], WebGestalt [73], and leapR [74]. Each of these databases highlight different biological activity—for example, KEGG is uniquely able to integrate metabolites with protein/gene-based markers, while the GO maps genes to subcellular compartments (among other things). Each tool also provides unique analysis such as rank-based enrichment (GSEA) or pairwise correlation analysis (leapR). Traditionally, proteogenomics datasets benefit from extensive prior knowledge databases, with defined IDs and known feature characteristics. As a result, current pathway analysis tools have been successful at helping build meaningful connections between features, facilitating new hypothesis generation. However, biological hypotheses are highly dependent upon the database used for the enrichment analysis, and the lack of a single database that can map all molecules (e.g. genes, proteins, metabolites, and especially lipids) is a large gap for multiomics analysis.

### Artificial intelligence is gaining traction in multiomics data integration

It is impossible to discuss multiomic integration without discussing how AI has impacted the field. Like most fields, integration of multiomic measurements has benefited from the recent breakthroughs in the usability of AI-based algorithms [75, 76]. One notable use of AI-based approaches is the integration of variational autoencoders as a method of reducing the dimensionality of complex multiomic data [77–79]. These tools provide alternatives to linear approaches such as principal component analysis or nonnegative matrix factorization. Additional AI advances have come from the incorporation of LLMs [80], which have been used to augment multiomics integration using clinical data [81] or information extracted from literature [82]. Lastly, foundation models built from libraries of single-cell or spatially resolved omics data enabled improved annotation of functional relationships between gene-based measurements [83, 84]. However, there are very limited applications of these approaches to small molecules other than basic chemical property prediction by tools such as ChemBERTa [85]. While newer AI-driven techniques show promise in expanding capabilities in multiomics data integration, their application is hindered by challenges such as limited explainability, reproducibility, and the absence of clear ground truth in multiomics data, making robust and reliable solutions difficult to achieve at present [86, 87]. There are also limitations with respect to the analysis objectives that are supported by AI-based tools, with most current examples performing predictions of external traits or assignment of subtypes (i.e. sample-focused analyses). Lastly, since AI/ML tools rely on the same pathway databases as other tools, they are also unable to use multiomic data to its full potential due to the lack of databases that accurately link all molecules, particularly lipids.

### Paucity of lipidomics support is a trend across all pathway tools

Across the examples we showed above, as well as the summary depicted in Fig. 2, there is a noticeable gap in the coverage for small molecular measurement for feature-focused analyses. While sample-focused tools such as DIABLO are able to identify metabolites and lipids of interest (Fig. 3A and B), the heavy reliance of feature-focused methods such as COSMOS on prior knowledge for connecting components from different -omics data in a meaningful way, together with the known difficulties in connecting lipids with other biological components makes it incredibly challenging to put these molecules in biological context [25]. This lack of support for integrating lipidomic data with other -omics data types is self-reinforcing, as this type of integration is an important mechanism for expanding and refining our understanding of how lipids interact with other components in biological systems, which in turn increases our ability to interpret and use lipidomic data both alone and in a multiomic context.

## Summary and perspective

### Metabolomic and lipidomic data integration can improve biological understanding

There is a significant gap in existing knowledge bases that support multiomic pathway analyses, significantly limiting the degree to which lipids can be meaningfully connected with other components in a coherent biological context. This gap is attributable to a variety of factors including the high degree of isomerism among lipid species measured in lipidomics, and a lack of clean mapping for complex lipid species onto existing cellular pathways. Ultimately, it is our view that functional integration of lipidomics data represents an area of profound opportunity within the broader field of multiomics for enhancing the depth and complexity of insights available from these studies.

**Figure 4:**
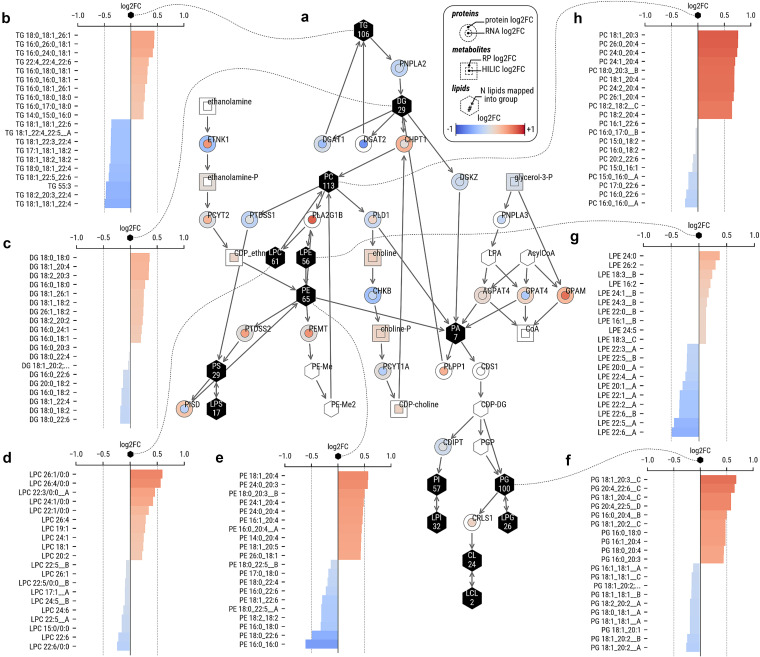
Manual multiomic mapping of protein, transcript, metabolite, and lipid measurements to a single pathway illustrates inherent complexities of small molecule pathway analysis. Log_2_ fold-change (log2FC) values calculated between the “Primitive” and “Committed” AML tumor classifications across transcriptomics, proteomics, metabolomics, and lipidomics measurements mapped to the “glycerolipids and glycerophospholipids” pathway from WikiPathways (https://www.wikipathways.org/pathways/WP4722). (a) Network visualization of pathway depicts round nodes as proteins, which may have corresponding transcript (outer ring) and/or protein (inner ring) log2FC values, square nodes represent metabolite log2FC values with corresponding HILIC measurement (inner circle), and/or RP measurement (outer ring), hexagon nodes represent lipid class measurements with counts of individual lipid species that map to each node included when available, and edges between all nodes represent directional relationships in the metabolic pathway (e.g. substrate to enzyme or enzyme to product). Log2FCs for individual lipid species representing the top 20 up- and down-regulated members of a lipid class are plotted around the margins of the network visualization for selected lipid classes: (b) triacylglycerols, TGs; (c) diacylglycerols, DGs; (d) lysophosphatidylcholines, LPCs; (e) phosphatidylethanolamines, PEs; (f) phosphatidylglycerols, PGs; (g) lysophosphatidylethanolamines, LPEs; and (h) phosphatidylcholines, PCs.

To further explore how the paucity of lipidomics data integration capabilities at the feature level can limit the ability to provide biological context to multiomic data, we performed an additional feature-focused analysis using the same AML cohort data from above. Specifically, we calculated the fold change between primitive and committed AML tumor classifications across all transcripts, proteins, metabolites, and lipids and then manually mapped the features to the “glycerolipids and glycerophospholipids” pathway from WikiPathways [46] (https://www.wikipathways.org/pathways/WP4722) (Fig. 4A). This pathway provides a high-level view of the synthesis and interconversion of major lipid classes and is among the most complete of the few existing reference pathways that meaningfully incorporates lipids with metabolites and proteins. The results are difficult to interpret: data from different modalities (e.g. transcriptomics versus proteomics in protein nodes) or measurement conditions (e.g. HILIC versus RP in metabolite nodes) are not always concurrent. Individual lipid species are typically mapped to larger LIPID MAPS subclasses [88], where there can be tens to hundreds of individual lipid species that can map to a given node (Fig. 4B–H), making meaningful interpretation even more difficult. Nevertheless, looking at the individual lipids within these groups, an interesting pattern does emerge: phosphatidylcholines (PCs, Fig. 4H), phosphatidylethanolamines (PEs, Fig. 4E), and phosphatidylglycerols (PGs, Fig. 4F) containing arachidonic acid (FA 20:4) are increased among primitive samples, whereas triacylglycerols (TGs, Fig. 4B), PEs (Fig. 4E), and lysophosphatidylethanolamines (LPEs, Fig. 4G) that contain polyunsaturated acids (PUFAs, e.g. FA 22:6) are increased among committed samples. While lipid-pathway annotation is necessary for lipidomic integration, this example shows that it is not sufficient: additional tools to interpret the different lipid subspecies and visualize them automatically (this figure was custom generated) are also lacking.

### The promise of network models, artificial intelligence, and pathway data curation

The challenges of multiomic data integration with lipidomics data stem from multiple factors including analytical capabilities, informatics pipelines for metabolite and lipid annotation from raw MS data, and the underlying libraries/database, statistics, and algorithms. While improving each of these aspects will improve overall small molecule interpretation, the multiomic tools that incorporate small molecules (Fig. 2) are currently insufficient. Therefore, an improvement in multiomics data integration tools to meaningfully incorporate lipids is the most impactful way to enable biological findings from these data.

One approach to improving the integration of multiomic data lies in biological networks [16, 89, 90]. Networks (or graphs) are a natural way to structure complex data made up of interactions (edges) between components (nodes). Heterogenous networks, or networks that contain multiple different node and/or edge types, are particularly well-suited for representing complex multiomics data without the information loss that inherently occurs when multiomics data is coerced into a uniform representation. Structuring multiomics data within a heterogenous network better supports the interpretation of multiomics data, both qualitatively through visualization methods and quantitatively through statistical methods and network-specific algorithms.

AI is another approach that could improve the integration of small molecule data. One major constraint we found in our example analysis above was the expectation of linear relationships between groups of functional similarly molecules. For example, enrichment statistics (Fig. 3B) generally seek out groups of molecules in a pathway that are differentially expressed in the same direction and with similar scales, implicating the pathway in the biological phenomenon measured. Deep learning algorithms, due to their founding in artificial neural networks, are easily able to identify nonlinear and indirect relationships between molecules [91]. Nonlinear modeling approaches could help capture the nuances of small molecule data in a combinatorial nature, such as the isomeric potential of metabolite measurements, and account for the various aspects of lipid structure, such as fatty acid composition, degree of unsaturation, or class-specific characteristics. Through its capacity to uncover hidden patterns that are difficult to detect using traditional methods, AI could provide insights into metabolite and lipid biology that significantly enhance functional analyses.

Lastly, both network integration and AI improvements will suffer the same fate as previous methods without improved molecular annotation and expanded knowledge bases. For example, most network modeling algorithms (e.g. Tuncbag *et al.* [92]) rely on often hand-curated relationship data between molecules, such as protein–protein interaction networks [93–95] or metabolic pathway information [66, 70]. AI, also, requires large training datasets upon which they can identify relationships in the data. As such, careful integration of lipidomic data to existing pathway models would dramatically help these tools move forward.

### Clinical and translational implications of improved lipidomics data integration

Recent studies demonstrate the tangible clinical impact of multiomics integration across diverse disease contexts. A proteogenomic survey of 242 ovarian tumors identified a panel of 1082 proteins that was able to predict response in patients high-grade serous ovarian cancer [96]. In a prospective precision oncology trial, the Tumor Profiler (TuPro) project applied nine independent -omics technologies to melanoma patient samples, generating comprehensive molecular profiles that informed treatment recommendations and achieved a 38% objective response rate in difficult-to-treat patients receiving individualized, multibiomarker-driven therapies over the standard of care [97].

Beyond direct treatment guidance, multiomics approaches have proven valuable for biomarker discovery and mechanistic insight, which could then inform drug development. Multiomics profiling of tumor-associated macrophages in esophageal squamous cell carcinoma uncovered a cholesterol-mediated immunosuppressive mechanism, identifying PLD3 expression as both a prognostic biomarker and potential therapeutic target [98]. Similarly, integrative analysis in HER2+ breast cancer revealed that the CNS-enriched metabolite N-acetylaspartate enables tumor immune evasion, suggesting its synthetic enzyme NAT8L as a target to enhance immunotherapy efficacy [99]. In Alzheimer’s disease, multiomics integration identified lysophosphatidylethanolamine (LPE) species, particularly LPE 22:6, as strongly associated with disease-relevant protein modules, positioning these lipids as both potential therapeutic targets and candidates for dietary supplementation strategies [100]. Notably, several of these clinically impactful insights either implicate specific lipid species directly or reveal mechanistic roles for lipid metabolism more broadly, even in cases where comprehensive lipidomics was not a primary focus of the investigation. This pattern suggests that lipid biology may be an underexplored contributor to disease mechanisms across diverse pathologies. As integration tools improve their capacity to systematically incorporate lipidomics data alongside other -omics layers, we anticipate that the landscape of clinically actionable biomarkers and therapeutic targets will expand substantially.

### Conclusions

In this work, we highlight the broad diversity of tools available for multiomics data integration and examine systematic gaps in tools that handle small molecules, particularly lipids. We identify numerous reasons for these gaps and propose potential solutions through graphical models, AI data curation, and improved pathway mapping. We conclude that better incorporation of lipids into pathway analyses is an essential next step in multiomics data integration tool development.

**Key Points**

- Multiomics analysis tools produce complex and complementary insights into biological processes.
- Multiomics data integration, especially pathway integration, is critical for translating complex multiomics data into meaningful biological insights.
- Different strategies for multiomics data integration exist that provide insights at the sample- or feature-level, and are best suited to different analysis objectives.
- Integration of small molecule -omics data faces unique barriers including isomerism among lipids and the lack of lipid representation in existing pathways.
- Promising solutions to these barriers include new data models for centralizing multimodal data, curation of reference pathways that include lipids, and application of artificial intelligence for predicting and scoring pathway dysregulation.

## Data Availability

Harmonized genomic, transcriptomic, proteomics, metabolomics, and lipidomics data files generated for the CPTAC AML cohort can be accessed via Genomic Data Commons (GDC) at: https://portal.gdc.cancer.gov.

## Notes

The research team is a group of postdoctoral researchers and senior scientists in the Biological Sciences Division at Pacific Northwest National Laboratory.
